# Putative rhamnogalacturonan-II glycosyltransferase identified through callus gene editing which bypasses embryo lethality

**DOI:** 10.1093/plphys/kiae259

**Published:** 2024-05-13

**Authors:** Yuan Zhang, Deepak Sharma, Yan Liang, Nick Downs, Fleur Dolman, Kristen Thorne, Ian M Black, Jose Henrique Pereira, Paul Adams, Henrik V Scheller, Malcolm O’Neill, Breeanna Urbanowicz, Jenny C Mortimer

**Affiliations:** Joint BioEnergy Institute, Emeryville, CA 94608, USA; Environmental Genomics and Systems Biology Division, Lawrence Berkeley National Laboratory, Berkeley, CA 94720, USA; Complex Carbohydrate Research Center, University of Georgia, Athens, GA 30602, USA; Department of Biochemistry and Molecular Biology, University of Georgia, Athens, GA 30602, USA; Joint BioEnergy Institute, Emeryville, CA 94608, USA; Environmental Genomics and Systems Biology Division, Lawrence Berkeley National Laboratory, Berkeley, CA 94720, USA; Joint BioEnergy Institute, Emeryville, CA 94608, USA; Environmental Genomics and Systems Biology Division, Lawrence Berkeley National Laboratory, Berkeley, CA 94720, USA; School of Agriculture, Food and Wine, University of Adelaide, Adelaide, SA 5005, Australia; Complex Carbohydrate Research Center, University of Georgia, Athens, GA 30602, USA; Department of Biochemistry and Molecular Biology, University of Georgia, Athens, GA 30602, USA; Complex Carbohydrate Research Center, University of Georgia, Athens, GA 30602, USA; Joint BioEnergy Institute, Emeryville, CA 94608, USA; Environmental Genomics and Systems Biology Division, Lawrence Berkeley National Laboratory, Berkeley, CA 94720, USA; Joint BioEnergy Institute, Emeryville, CA 94608, USA; Environmental Genomics and Systems Biology Division, Lawrence Berkeley National Laboratory, Berkeley, CA 94720, USA; Joint BioEnergy Institute, Emeryville, CA 94608, USA; Environmental Genomics and Systems Biology Division, Lawrence Berkeley National Laboratory, Berkeley, CA 94720, USA; Department of Plant and Microbial Biology, University of California, Berkeley, CA 94720, USA; Complex Carbohydrate Research Center, University of Georgia, Athens, GA 30602, USA; Complex Carbohydrate Research Center, University of Georgia, Athens, GA 30602, USA; Department of Biochemistry and Molecular Biology, University of Georgia, Athens, GA 30602, USA; Joint BioEnergy Institute, Emeryville, CA 94608, USA; Environmental Genomics and Systems Biology Division, Lawrence Berkeley National Laboratory, Berkeley, CA 94720, USA; School of Agriculture, Food and Wine, University of Adelaide, Adelaide, SA 5005, Australia

## Abstract

Rhamnogalacturonan II (RG-II) is a structurally complex and conserved domain of the pectin present in the primary cell walls of vascular plants. Borate cross-linking of RG-II is required for plants to grow and develop normally. Mutations that alter RG-II structure also affect cross-linking and are lethal or severely impair growth. Thus, few genes involved in RG-II synthesis have been identified. Here, we developed a method to generate viable loss-of-function *Arabidopsis* (*Arabidopsis thaliana*) mutants in callus tissue via CRISPR/Cas9-mediated gene editing. We combined this with a candidate gene approach to characterize the *male gametophyte defective 2* (*MGP2*) gene that encodes a putative family GT29 glycosyltransferase. Plants homozygous for this mutation do not survive. We showed that in the callus mutant cell walls, RG-II does not cross-link normally because it lacks 3-deoxy-D-manno-octulosonic acid (Kdo) and thus cannot form the α-L-Rha*p*-(1→5)-α-D-kdo*p*-(1→sidechain). We suggest that *MGP2* encodes an inverting *R*G-II *C*MP-β-*K*do *t*ransferase (RCKT1). Our discovery provides further insight into the role of sidechains in RG-II dimerization. Our method also provides a viable strategy for further identifying proteins involved in the biosynthesis of RG-II.

## Teaser

Development of a gene-editing method in callus that bypasses embryo lethality reveals an essential gene responsible for adding sugar to the conserved pectic domain in the primary cell walls of plants.

## Introduction

The primary wall surrounding growing plant cells is a dynamic structure comprised primarily of cellulose, hemicellulose, and pectin (Cosgrove 2022). The synthesis of these polysaccharides involves a large number of genes, which together may account for up to 10% of a plant's genome. The *Arabidopsis* (*Arabidopsis thaliana*) genome encodes at least 567 predicted glycosyltransferases (GTs) across 44 carbohydrate-active enzyme (CAZy) families. Many of these GTs are involved in cell wall glycan synthesis. However, only a small number of them have been functionally characterized (Ulvskov and Scheller 2018).

**Figure 1.**
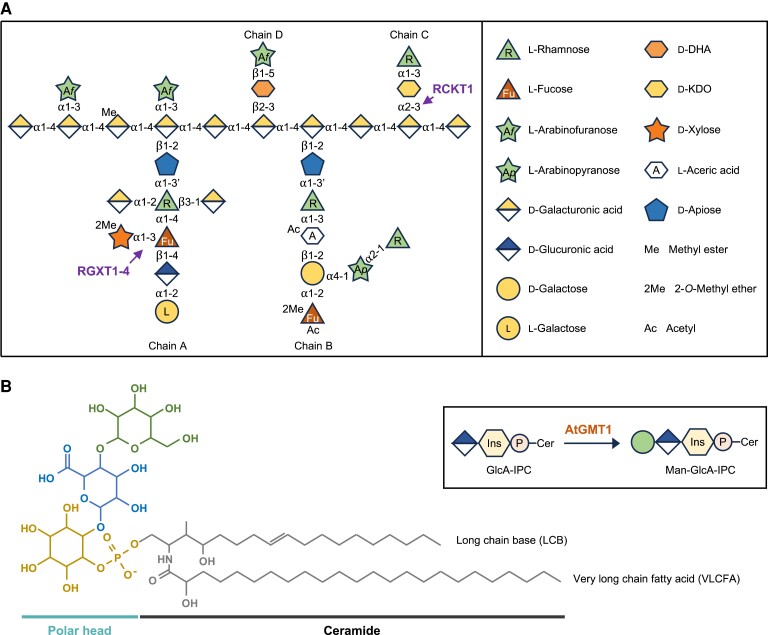
Structure of RG-II and GIPC. **A)** Schematic structure of RG-II (modified from Sechet *et al.* 2018). Monosaccharides are shown using the symbol nomenclature for glycans (Varki et al. 2015; Neelamegham et al. 2019). **B)** Example structure of a GIPC showing the ceramide (black), inositol phosphate (yellow), GlcA (blue), and mannose (Man, green) (modified from Mortimer and Scheller 2020). Man-GlcA-IPC is the dominant headgroup structure in wild-type *Arabidopsis* callus tissue. Inset: the synthesis of Man-GlcA-IPC catalyzed by *Arabidopsis* GMT1 (AtGMT1). Man and GlcA are shown using the same symbol nomenclature for glycans as in **A)**.

Rhamnogalacturonan II (RG-II) is a structurally complex and conserved domain of the pectin present in the primary cell walls of all vascular plants. Borate cross-linking of this RG-II is required for these plants to grow and develop normally (Kobayashi et al. 1996; O’Neill et al. 2004; Bar-Peled et al. 2012; González-Fontes 2020; Yang and Anderson 2020). Mutations that result in alterations to RG-II structure also affect cross-linking and may be lethal or severely impair growth (O’Neill et al. 2001; Delmas et al. 2008; Reboul et al. 2011; Pabst et al. 2013; Rautengarten et al. 2016; Qi et al. 2017; Sechet et al. 2018). Thus, few genes involved in RG-II synthesis have been identified (Ishii and Matsunaga 1996; Ishii et al. 2001; Matsunaga et al. 2004; Funakawa and Miwa 2015). RG-II has a backbone of homogalacturonan (HG) that is decorated with 4 structurally distinct sidechains (A to D) and 2 arabinofuranosyl (Ara*f*) substituents (Fig. 1A). The sidechains are composed of 12 different monosaccharides linked by at least 20 different glycosidic bonds. Thus, RG-II is the most structurally complex polysaccharide yet identified in nature (O’Neill et al. 2004; Scheller et al. 2006; Mohnen 2008; Ndeh et al. 2017).

Plants carrying mutations in genes affect RG-II synthesis and structure typically exhibit severe growth defects. For example, the *Arabidopsis murus 1* (*mur1*) mutant, which is impaired in its ability to synthesize GDP-L-fucose (Fuc), is unable to form a normal Sidechain A and has a dwarf phenotype (O’Neill et al. 2001; Pabst et al. 2013). Similar phenotypes were obtained by silencing the major Golgi-localized GDP-L-Fuc transporter in the *gft1* mutant (Rautengarten et al. 2016) and by mutations affecting the synthesis and transport of other monosaccharides required to form RG-II, including GDP-L-galactose (Gal) (Qi et al. 2017; Sechet et al. 2018); UDP-D-glucuronic acid (GlcA), a precursor for UDP-D-apiose (Api) (Reboul et al. 2011); UDP-D-Api/UDP-D-xylose (Xyl) (Ahn et al. 2006); and CMP-β-3-deoxy-D-manno-octulosonic acid (Kdo) (Delmas et al. 2008).

Identifying the GTs involved in the formation of RG-II is required to understand the role of this polysaccharide in cell wall integrity and plant growth. Reverse genetic approaches have had limited success in identifying RG-II-specific GTs. This is primarily due to the deleterious effects that even minor modifications to RG-II structure have on plant growth. To date, only 2 GTs have been implicated in RG-II synthesis. Rhamnogalacturonan xylosyltransferases (RGXT) 1 to 4, a family of GTs is believed to add Xyl to Sidechain A (Egelund et al. 2006; Egelund et al. 2008; Petersen et al. 2009; Liu et al. 2011). Galacturonosyltransferase 4 (GAUT4) may be responsible for forming the HG backbone (Biswal et al. 2018). Loss-of-function mutations in GAUT4 and RGXT4 are lethal. Thus, alternate strategies were used to gain insight into their functions: RNA interference knockdown of *GAUT4* in switchgrass and poplar caused a reduction in the amounts and cross-linking of RG-II (Biswal et al. 2018); a pollen-rescue strategy enabled the generation of a homozygous RGXT4 (also known as male gametophyte defective 4 [MGP4]) loss-of-function mutant, which showed decreased Xyl content and reduced RG-II dimerization (Liu et al. 2011). Nevertheless, both strategies have their limitations. Knockdown of target gene expression may be insufficient and obscure the consequences of gene loss of function. The pollen rescue strategy is effective only if lethality is associated with the defective male gametophyte. Both methods require extensive crossbreeding and screening yet offer no assurance of achieving stable lines. Hence, there is a need to establish a facile system to generate viable plant lines lacking the expression of functional GTs.

Glycosylinositol phosphorylceramides (GIPCs) are an important class of plasma membrane lipids in plants (Fig. 1B) (Mortimer and Scheller 2020). Previously, we observed that plants carrying mutations that affect the glycosylation of GIPCs had severe growth defects, yet their null mutant calli grew well (Mortimer et al. 2013; Fang et al. 2016; Ishikawa et al. 2018). GIPCs have been proposed to have a role in cell–cell adhesion (Singh et al. 2005; Ishikawa et al. 2018), wherein RG-II may also be involved, given its contribution to the formation of the pectin network through borate cross-linking. For example, reduced RG-II cross-linking in the *mur1* mutant results in defective cell adhesion, specifically at the interface between stele and cortex cells (Voxeur et al. 2017). The *rotten ear* maize mutant also exhibits decreased cell adhesion as well as disrupted cell wall organization in the ear, which has been attributed to incomplete borate cross-linking of RG-II (Chatterjee et al. 2014). This led us to hypothesize that genetically edited callus tissue may provide an opportunity to identify genes responsible for glycan synthesis that are harmful to normal plant growth and development. Thus, we developed a CRISPR-mediated tissue culture transformation approach to generate callus carrying loss-of-function GT mutations. We validated this approach with the well-characterized GIPC mannosyltransferase 1 (AtGMT1) (Fig. 1B) (Fang et al. 2016). We then generated a loss-of-function mutant of a CAZy family GT29 protein (MGP2), which is currently annotated as a sialyltransferase (ST)-like protein in The Arabidopsis Information Resource (TAIR) (arabidopsis.org). The callus mutant together with NMR spectroscopic and mass spectrometric analysis of its RG-II provided evidence that MGP2 is the CMP-β-Kdo transferase (RCKT1) required to form the α-L-Rha*p*-(1→5)-α-D-Kdo*p*-(1→sidechain of RG-II).

## Results

### *Arabidopsis* GT29 family members are highly coexpressed with primary cell wall- and GIPC-related genes

To identify GTs implicated in the cell–cell adhesion process, we used bioinformatics and publicly available arrays of 113 developmental RNA-seq data sets, representing 11 distinct tissue and organ types to identify genes that are coexpressed with *Arabidopsis GMT1* (Jarvis et al. 2003; Roberts and Gonzalez-Carranza 2007). Disrupting this gene causes severe plant growth defects and reduces cell–cell adhesion (Singh et al. 2005; Fang et al. 2016). We then used SUBA (Subcellular Location Database for *Arabidopsis* Proteins) to refine our list to 75 candidates with likely Golgi localization (Supplementary Data Set S1). These candidates were regarded as putative GTs relevant to cell–cell adhesion. Somewhat unexpectedly, all 3 *Arabidopsis* family GT29 members—At1g08280 (GALT29A), At1g08660 (RCKT1, also known as MGP2), and At3g48820 (SIA2)—were included. They are annotated as ST-like proteins since they possess motifs characteristic of mammalian family GT29 STs. However, the plant enzymes have no discernible ST activity (Daskalova et al. 2009). This is not entirely unexpected since plants produce little if any sialic acid (Zeleny et al. 2006).

We then considered the possibility that the plant GT29 enzymes use a nucleotide sugar donor substrate structurally related to CMP-sialic acid, the donor for STs. Indeed, *RCKT1* and *SIA2* have been proposed to encode enzymes that catalyze the transfer of 2-keto-3-deoxy-D-manno-octulosonic acid (Kdo) or 2-keto-3-deoxy-D-lyxo-heptulosaric acid (Dha) (Angata and Varki 2002; Paccalet et al. 2007; Deng et al. 2010; Dumont et al. 2014). The activated form of Dha remains to be identified. CMP-β-Kdo is the activated form of Kdo, and the family GT29 proteins utilize an inverting mechanism for glycosyl transfer, indicating they would be able to form the α-2,3 bond that links Kdo to the RG-II backbone and initiates the formation of Sidechain C (Fig. 1A).

### Creating biallelic callus mutants carrying GT knockouts

**Figure 2.**
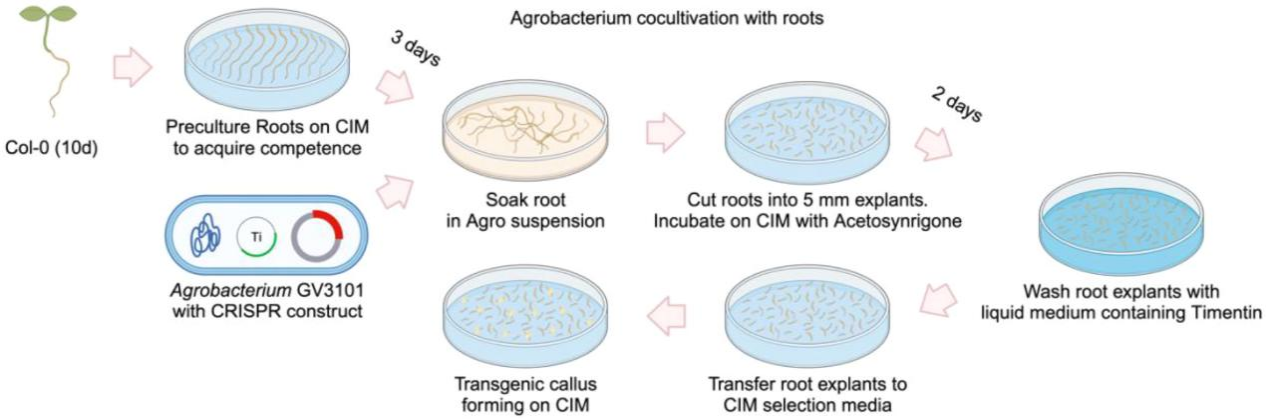
*Agrobacterium*-mediated root transformation of *Arabidopsis*. Schematic illustration of the transformation procedure of *Arabidopsis* root system to generate transgenic callus. Primary roots are taken from 10-d-old *Arabidopsis* seedlings and precultured on CIM. After 3 d, the primary roots are infected with Agrobacteria carrying the T-DNA vector and then cut into root explants. The infected root explants are incubated on cocultivation media for 2 d and then washed using liquid media supplemented with timentin (100 mg/L) to neutralize the infecting Agrobacteria. The explants are then incubated on CIM supplemented with timentin and kanamycin, for the selection of transgenic callus with stable T-DNA integration, which usually form within 1 mo (figure created using BioRender; BioRender.com).

No homozygous mutants of *GALT29A*, *RCKT1* (*MGP2*), and *SIA2* have been isolated, suggesting they are lethal or severely harm the mutant plants (Deng et al. 2010; Dumont et al. 2014). This also suggests that these GTs have an essential role in plant development. This led us to develop a CRISPR/Cas9-mediated tissue culture transformation strategy to generate loss-of-function callus to study the function of GTs. CRISPR/Cas9 technology is a powerful tool for targeted genome editing in plants and has considerable potential for generating knockout mutants. Here, we optimized a tissue culture-based transformation method (Vergunst et al. 1998) and used it to introduce the CRISPR/Cas9 system with the aim of circumventing the need to produce whole plants and viable seeds (Fig. 2). The isolated clonal knockout lines can be indefinitely propagated as callus tissue, which provides material suitable for biochemical characterization of the altered primary cell wall.

**Figure 3.**
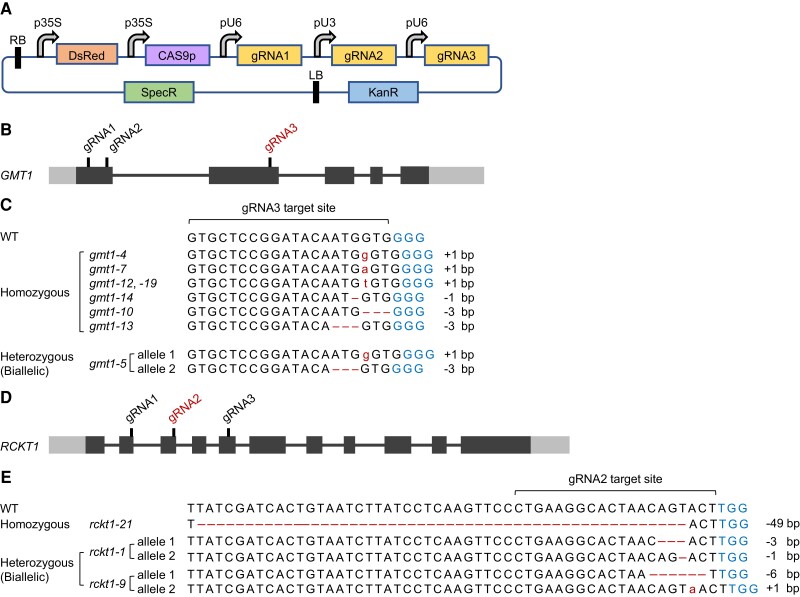
Creation of CRISPR/Cas9-edited *Arabidopsis* callus lines with different mutations on *GMT1* and *RCKT1*. **A)** Illustration of the T-DNA transformation vector used for CRISPR editing of the target gene. *Arabidopsis* U6 (pU6) promoter is used to express gRNA1 and gRNA3, while *Arabidopsis* U3 (pU3) promoter is used to express gRNA2. **B)** Schematic diagram of the *GMT1* gene structure with gRNA-targeting sites labeled. The black boxes represent exons; the gray boxes represent 5′-UTR and 3′-UTR; the black lines represent introns. CRISPR/Cas9 editing was only detected at the gRNA3 (red) target site. **C)** Gene-editing events at the gRNA3 target site of the *gmt1* callus mutants. Seven homozygous lines were identified: 5 are *gmt1* knockout mutants with frameshifted and truncated GMT1 protein, and 2 are mutants with the loss of a single amino acid from the GMT1 protein sequence. One heterozygous/biallelic line was identified with 2 mutant alleles, one with a 1-bp insertion causing truncation of the GMT1 protein and the other with a 3-bp deletion resulting in the loss of 1 amino acid from the GMT1 protein sequence. The words in red color denote indel mutations, and the words in blue color denote the protospacer adjacent motif (PAM) sequences. **D)** Schematic diagram of the *RCKT1* gene structure with gRNA-targeting sites labeled. CRISPR/Cas9 editing was only detected at the gRNA2 (red) target site. **E)** Gene-editing events at the gRNA2 target site of the *rckt1* callus mutants. The homozygous knockout mutant *rckt1-21* has a 49-bp deletion within the *RCKT1* gene, creating a frameshifted and truncated RCKT1 protein. The *rckt1-1* heterozygous/biallelic line has 2 mutant alleles, one with a 3-bp deletion causing the loss of 1 amino acid from the RCKT1 protein and the other with a 1-bp deletion resulting in frameshift and premature stop codon in the protein sequence. The *rckt1-9* heterozygous line has 2 mutant alleles, one with a 6-bp deletion causing the loss of 2 amino acids from the RCKT1 protein and the other with a 1-bp insertion resulting in frameshift and premature stop codon in the protein sequence. The words in red color denote indel mutations, and the words in blue color denote the PAM sequences. DsRed, DsRed fluorescence reporter; KanR, kanamycin resistance marker; LB, left border region; p35S, cauliflower mosaic virus (CaMV) 35S promoter; pU3, *Arabidopsis* ubiquitin3 promoter; pU6, *Arabidopsis* ubiquitin6 promoter; RB, right border region; SpecR, spectinomycin resistance marker.

We used a modular cloning system tailored from Lowder et al. (2015), which includes a T-DNA destination vector, a Cas9 entry vector, and a guide RNA (gRNA) entry vector (Supplementary Fig. S1). To increase the rate of editing, each construct contained 3 single guide RNA (sgRNAs) for the target gene (Fig. 3A; Supplementary Table S1). Since CRISPR/Cas9 editing typically generates small insertions and deletions (indels) through nonhomologous end joining, the disruption of gene function primarily stems from frameshift mutations. To maximize the impact of such disruptions, the sgRNAs were designed to target the protein-coding region proximal to the 5′-UTR.

### Validating the system with a known GT as the target for knockout

We first targeted *AtGMT1* to test the effectiveness of our CRISPR/Cas9 toolbox and tissue culture transformation method in creating GT knockout mutant calli. We targeted 3 unique sites in *AtGMT1*: 2 in Exon1 and 1 in Exon2 (Fig. 3B). The primary roots of 10-d-old *Arabidopsis* seedlings were used for *Agrobacterium*-mediated transformation, yielding 16 viable calli out of 300 infected root explants (5.3%). The stable integration of the CRISPR/Cas9 cassette in these transformants was indicated by their resistance to kanamycin and the detection of DsRed fluorescence and further validated via PCR.

To characterize the editing events in each transformant, the genomic region spanning the 3 target sites was amplified by PCR, Sanger sequenced, and then analyzed using DECODR v3.0 (https://decodr.org/) (Bloh et al. 2021). Cas9-induced mutations were introduced into 13 lines (81%) at the sgRNA3 target site in Exon2. This gave us 7 homozygous mutants (with 2 identical mutant alleles), 1 biallelic mutant (with 2 distinct mutant alleles), and 5 chimeric mutants (carrying 2 or more types of alleles, with or without mutation) (Fig. 3C). The 7 editing events detected in the homozygous lines consisted of 5 frameshifting indel mutations, expected to result in truncated proteins, and 2 in-frame deletion mutations that would lead to the loss of 1 amino acid (Fig. 3C). The single biallelic mutant had 1 allele with a frameshift insertion and another with a frame-preserving deletion (Fig. 3C). All the transgenic lines were unedited at the sgRNA1 and sgRNA2 target sites of Exon1, while 3 lines completely escaped targeted editing of *AtGMT1* by the CRISPR/Cas9 complex. Thus, our strategy enables the efficient creation of clonal knockout lines in the form of callus, where every individual cell carries the same frameshift mutation within the target gene.

### Validating the function of *gmt1* by GIPC analysis

We analyzed the GIPCs of the callus lines to assess the suitability of homozygous frameshift callus mutants for decreasing or eliminating GT activity. A transgenic callus line without CRISPR editing was included as a nonedited (NE) control. We also examined callus derived from Col-0 seedling roots and a *gmt1-3* T-DNA insertional mutant.

GIPCs were extracted from callus tissue grown in liquid media, and the degree of glycosylation was determined using thin-layer chromatography (TLC) (Jing et al. 2021). The GIPC profiles of the *gmt1-4* and *gmt1-3* mutants were clearly different from those of Col-0 and the NE control (Supplementary Fig. S2). Notably, the GIPC bands exhibited a larger shift in both *gmt1* mutants, indicating a reduction in molecular weight due to the absence of mannosylation (Fang et al. 2016). Furthermore, the bands displayed a compact and tightly clustered appearance in contrast to the dispersed bands observed in the negative control lines. This distinctive feature marked a substantial decrease in the diversity of GIPC species when normal mannosylation of the sugar head group is absent.

We next determined the mannose content of the extracted GIPCs. The monosaccharides were released by hydrolysis with trifluoroacetic acid (TFA) and quantified by high-performance anion exchange chromatography coupled with pulsed amperometric detection (HPAEC-PAD) (Fang et al. 2016). The mannose content was substantially reduced (3- to 7-fold) in both *gmt1* mutants compared to that in the wild-type and NE control (Supplementary Table S2). Our qualitative TLC assay and glycosyl residue composition analysis demonstrate that the *gmt1* CRISPR callus mutant recapitulates the GPIC chemotype of the *gmt1-3* T-DNA insertional mutant.

### Creating homozygous *RCKT1* knockout callus mutants

We next targeted the *RCKT1* gene identified in our bioinformatic screen. The tri-gRNA module in the transformation vector was designed to target 3 distinct sites within the *RCKT1* coding region (Exons 2, 3, and 5) (Fig. 3D). We obtained 16 viable calli out of 410 infected root explants (3.9% efficiency). Cas9-mediated editing was restricted to the sgRNA2 target site of *RCKT1*. Genotyping of the transgenic lines identified 1 homozygous mutant, 2 heterozygous/biallelic mutants, 11 chimeric mutants, and 1 NE control line. The homozygous mutant *rckt1-21* carried a “−49 bp/−49 bp” biallelic mutation, disrupting the ORF and introducing an early stop codon (Fig. 3E). Consequently, the predicted product of the *RCKT1* mutant allele in *rckt1-21* is a truncated protein consisting of 104 amino acid residues at the N-terminus, with 73 residues preserved and 31 altered. This mutant RCKT1 variant is expected to lack the catalytic domain, rendering *rckt1-21* an ideal knockout line for cell wall analysis. In contrast, the 2 heterozygous mutants, *rckt1-1* and *rckt1-9*, harbored a “−1/−3” and “+1/−6” biallelic mutation, respectively (Fig. 3E). These mutations resulted in 1 allele carrying a frameshift indel mutation (−1 or +1), while the other allele containing a frame-preserving deletion (−3 or −6), which led to the loss of 1 or 2 amino acids. It was unknown whether such biallelic mutations would cause the complete loss of *RCKT1* function in these heterozygous mutants. The chimeric mutants possessed wild-type alleles at varying percentage (6% to 52%), which remained recalcitrant to CIRSPR/Cas9 editing.

The homozygous *rckt1-21* and the heterozygous/biallelic *rckt1-1* and *rckt1-9* are gene-edited lines that can be used to study AtRCKT1 function. The NE *rckt1-6*, carrying the T-DNA insertion but lacking any *AtRCKT1* editing, was included as a control.

### The absence of RG-II Kdo from the *rckt1* callus mutants

**Figure 4.**
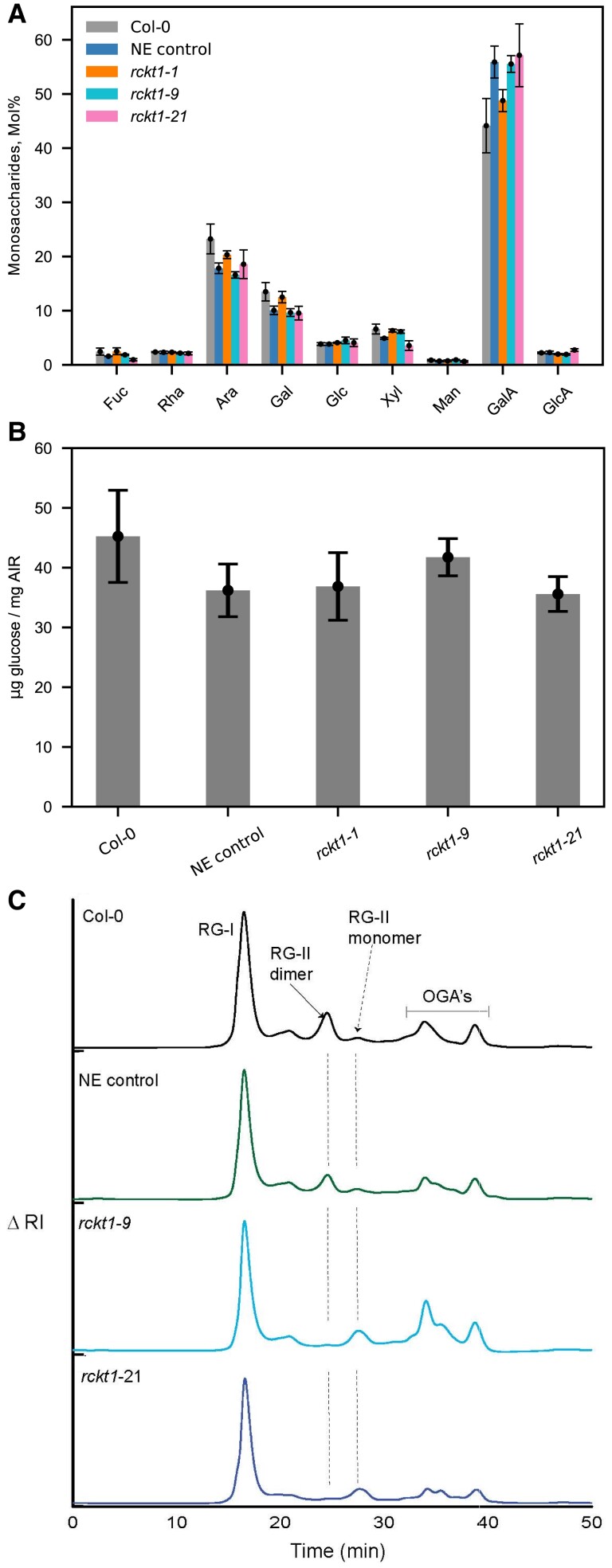
Cell wall polysaccharides and pectin analysis of *rckt1* callus mutants. **A)** Monosaccharide composition of noncellulosic cell wall polysaccharides **B)** Cellulose-derived glucose. Data in **A)** and **B)** are means of 3 biological replicates ± Sd. No statistically significant difference (ANOVA test) was observed between Col-0 wild type, NE control, and the homozygous and heterozygous/biallelic *rckt1* mutants. **C)** Separation of pectic domains using SEC. The representative SEC profiles of EPG and PME treated AIRs are shown for Col-0 wild type (black), NE Control (green), *rckt1-9* (light blue), and *rckt1-21* (dark blue). The position of RG-I, RG-II dimer and monomer, and the oligogalacturonides are shown.

To explore the effect of *RCKT1* mutations on cell wall glycosyl residue composition, we grew the *rckt1* callus lines and controls in liquid media and then isolated their cell walls as alcohol-insoluble residue (AIR) for analysis. The monosaccharide compositions of the walls from the *rckt1* mutants were similar to those from the wild-type and the NE control lines (Fig. 4, A and B; Supplementary Data Set S2). However, we were unable to detect Kdo in intact AIR; thus, we isolated RG-II from the callus walls. To this end, we treated the AIR with an endopolygalacturonase (EPG) and separated the solubilized products by size-exclusion chromatography (SEC) (Barnes et al. 2021) to obtain an RG-II-rich fraction (Fig. 4C). This procedure also shows the relative abundance of the RG-II monomer and dimer present in the walls at the time of extraction. The dimer accounts for ∼80% of the RG-II in Col-0 and the NE control (Fig. 4C; Supplementary Table S3), a result consistent with previous reports (Ishii et al. 2001; Reuhs et al. 2004; Sechet et al. 2018). However, in both the homozygous *rckt1-21* mutant and the heterozygous/biallelic *rckt1-9* mutant, the monomer accounted for at least 74% of the RG-II (Fig. 4C; Supplementary Table S3), showing that the ability to form the dimer is diminished in the *rckt1* mutants.

**Figure 5.**
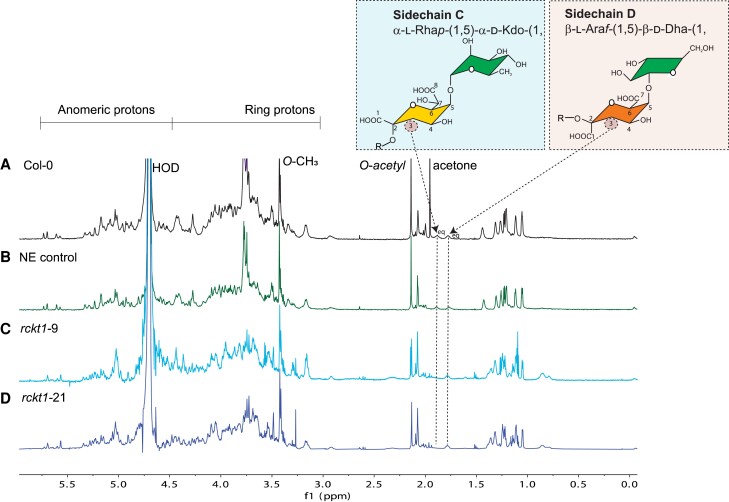
^1^H-NMR analysis of RG-II. RG-II (dimers) was isolated from the callus cell wall of **A)** Col-0 wild-type, **B)** NE control, **C)***rckt1-9*, and **D)***rckt1-21* and subsequently treated with dilute HCl to generate monomers for analysis. The diagnostic resonance of 2-keto-3-deoxy-D-manno-octulosonic acid (Kdo) is shown in **A)** (black) and **B)** (green) but absent from **C)** (light blue) and **D)** (dark blue). The 2-keto-3-deoxy-D-lyxo-heptulosaric acid (Dha) signal is detected in all the samples. The chemical structures of Sidechains C and D are shown in the blue and orange insets, respectively. The proton present at the equatorial positions at the third carbon of Kdo and Dha, respectively, is denoted as eq.

**Figure 6.**
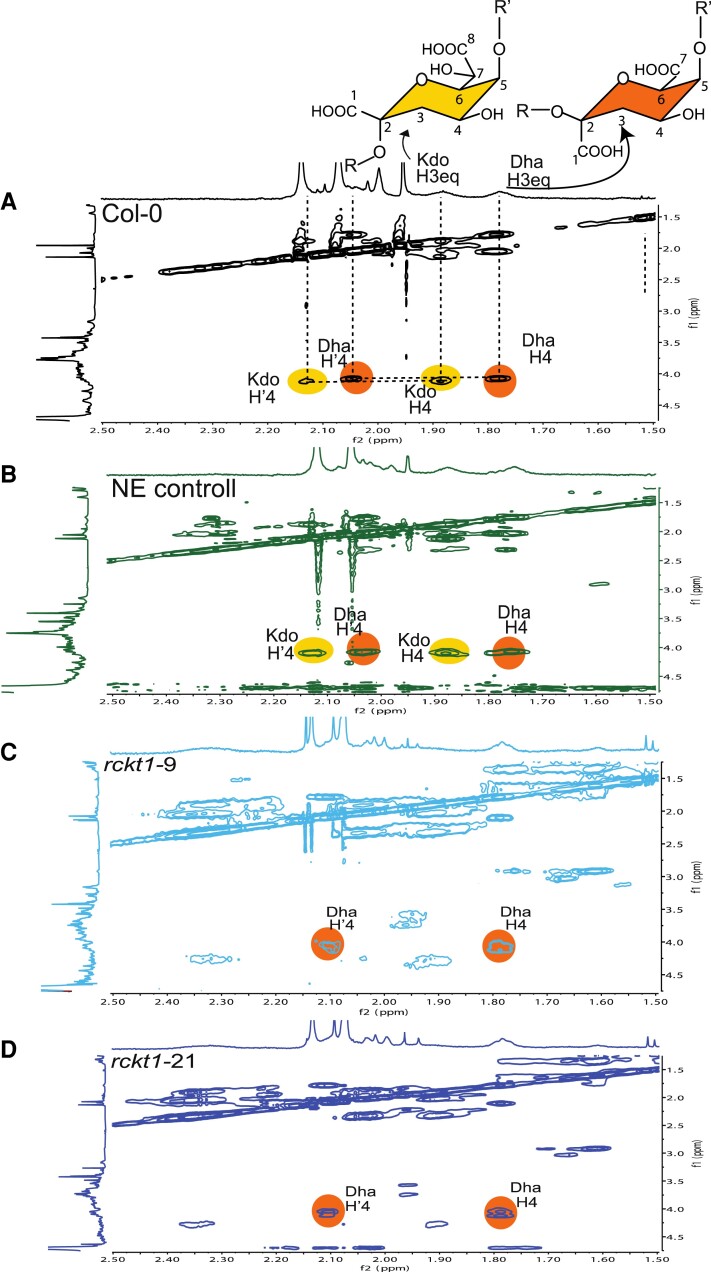
2D TOCSY NMR analysis of RG-II. RG-II (dimers) was isolated from the callus cell wall of **A)** Col-0 wild type, **B)** NE control, **C)***rckt1-9*, and **D)***rckt1-21* and subsequently treated with dilute HCl to generate monomers for analysis. The diagnostic resonance of 2-keto-3-deoxy-D-manno-octulosonic acid (Kdo) highlighted in yellow is shown in **A)** (black) and **B)** (green) but absent from **C)** (light blue) and **D)** (dark blue). The 2-keto-3-deoxy-D-lyxo-heptulosaric acid (Dha) signal highlighted in orange is detected in all the samples. The chemical structure of Kdo (yellow) and Dha (orange) are shown on the top.

The absence of Kdo in the RG-II from the 2 *rckt1* mutants was first established by solution-state NMR spectroscopy. To facilitate the detection of signals corresponding to Kdo and Dha, the RG-II (dimer) was treated with dilute HCl to generate the monomer. Resonances diagnostic for Kdo (H3, δ 1.88 ppm) and Dha (H3, δ 1.78 ppm) were clearly discernible in the wild-type RG-II (Fig. 5, A and B). To the contrary, the Kdo signal was absent in the *rckt1* mutants (Fig. 5, C and D). A 2D total correlation spectroscopy (TOCSY) NMR experiment showed that only the wild-type RG-II contained resonances characteristic of H3 and H3′ (δ 1.88 and 2.13 ppm) associated with H4 (δ 4.13) of Kdo, whereas the Dha H3 and H3′ resonance (δ 1.78 and 2.05 ppm) associated with H4 (δ 4.08) were present in RG-II from both the controls and the *rckt1* mutants (Fig. 6).

We next prepared the trimethylsilyl (TMS) methyl ester methyl glycoside derivatives of the RG-IIs for analysis by gas chromatography with electron impact MS (GC-EI-MS) (Barnes et al. 2021). Dha is present in the controls and the *rckt1* mutants, whereas Kdo is detected in the controls, but absent in the *rckt1-21* homozygous mutant heterozygous/biallelic mutant (Supplementary Fig. S3). In summary, our NMR spectroscopic and glycosyl residue composition analyses demonstrate that the homozygous mutant and the *rckt1-9* heterozygous/biallelic mutant produce RG-II that lacks discernible amounts of Kdo. This evidence supports our hypothesis that RCKT1 is the Kdo transferase required to form RG-II Sidechain C.

### Predicted structure of RCKT1 in the presence of CMP-β-Kdo

**Figure 7.**
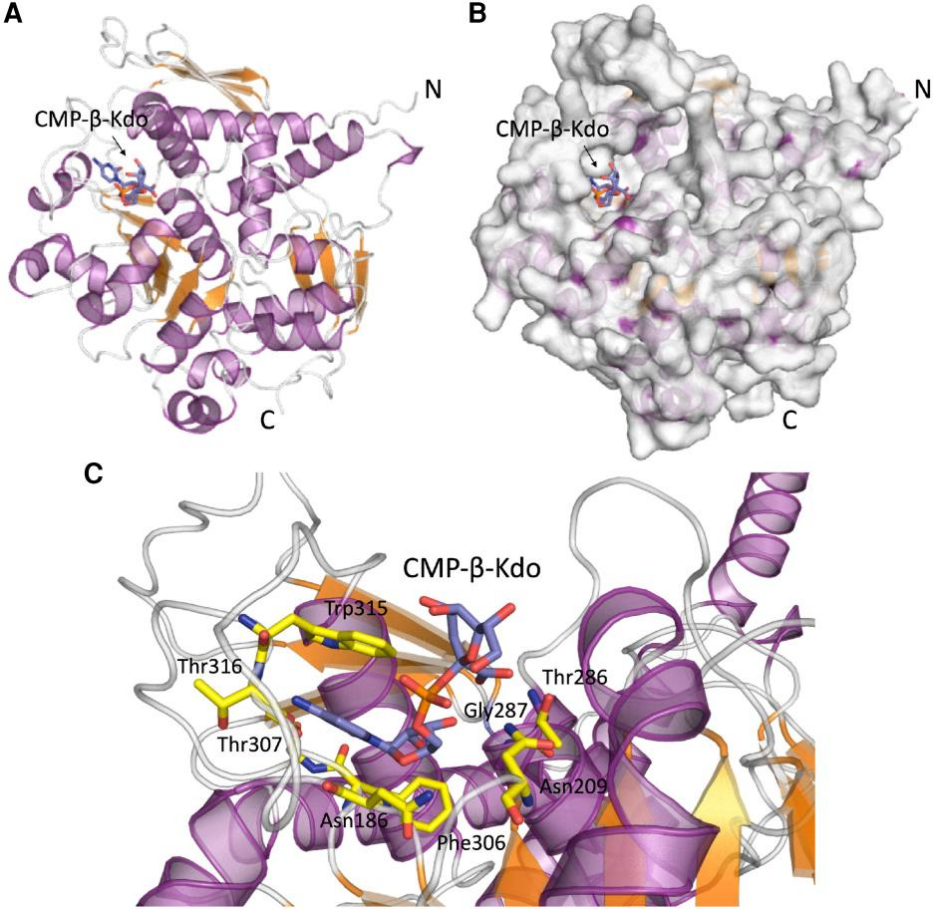
Structure of *Arabidopsis* RCKT1 predicted by AlphaFold modeling. **A)** Cartoon representation of general architecture of RCKT1 model. The nucleotide donor substrate, CMP-β-Kdo (blue), is positioned atop a central β-sheet comprising 7 parallel β-strands (orange) surrounded by 14 ɑ-helices (purple) and 2 small β-sheets (orange). **B)** Surface representation of RCKT1 structure in complex with CMP-β-Kdo (blue). It reveals the potential CMP-β-Kdo binding site and the surrounding binding cleft, which is a likely location for the binding of the acceptor substrate. **C)** Zoom in on the CMP-β-Kdo (blue) binding site showing all the residues (yellow) interacting via hydrogen bond and aromatic interactions between RCKT1 and CMP-β-Kdo ligand.

Determining the biochemical properties of RCKT1 is challenging since its acceptor is unknown and the CMP-β-Kdo donor has an estimated half-life of ∼34 min (Sugai et al. 1995; Wen et al. 2016). Thus, we used AlphaFold (Jumper et al. 2021) to predict the conformation of RCKT1 and gain some molecular insights into its function. The model shows an N-terminal transmembrane domain from residues 1 to 36 with the transmembrane ɑ-helix between residues 12 and 36. RCKT1 is a mix of ɑβ folds, comprising 1 central β-sheet consisting of 7 parallel β-strands surrounded by 14 ɑ-helices and 2 small β-sheets (Fig. 7, A and B). The mammalian α-2,3-ST ST3Gal-I (PDB ID 2WNB) from *Sus scrofa* (wild pig) (Rao et al. 2009) shares the highest amino acid sequence identity (32%) with RCKT1 in the Protein Data Bank. Both enzymes are members of the GT29 family, whose structurally characterized members have been shown to adopt a modified GT-A fold, but lack the characteristic DxD motif (Meng et al. 2013). The structure of the GT42 ST CstII (PDB ID 1RO7) from *Campylobacter jejuni* (Chiu et al. 2004), which has similar folding to RCKT1, was solved in complex with CMP-3FNeuAc. A superposition of CStII and RCKT1 allowed us to identify the likely position of the CMP binding site and consequently model CMP-β-Kdo into the substrate donor active site of RCKT1. This putative binding site suggests that the main and sidechain atoms of Phe306, Thr307, and Thr316 directly contact the cytidine nucleotide. Moreover, Trp315 makes aromatic stacking interactions. The ribose portion of the substrate interacts with the main chain N atoms of Asn186 and Gly287, and the Kdo sugar region showed 2 hydrogen bonds with Thr236 (Fig. 7C). It will be important to test the importance of these predicted residues in the future structural study.

## Discussion

### RG-II structure integrity is important for its function

It is widely recognized that the structure of RG-II must be maintained to ensure its normal dimerization, which is a key factor in its functions related to plant growth, development, and viability. This perspective has primarily been supported by research on plant mutants deficient in RG-II-specific GTs, enzymes, and transporters responsible for RG-II-specific nucleotide sugars, as well as boron uptake/transport mutants. Knockdown of the RGXTs that are involved in Sidechain A formation (Liu et al. 2011) and disruption of the synthesis and transport of RG-II nucleotide sugars, such as GDP-L-Fuc (O’Neill et al. 2001; Pabst et al. 2013; Rautengarten et al. 2016), GDP-L-Gal (Voxeur et al. 2011; Qi et al. 2017; Sechet et al. 2018), and UDP-D-Api (Reboul et al. 2011), all led to severe growth and developmental defects in plants. The truncation of Sidechain A, due to the absence of D-Xyl, L-Fuc, L-Gal, or D-Api, is believed to be responsible for the defective RG-II dimerization. These data are consistent with the notion that the borate cross-link formed between 2 RG-II monomers involves the chain A via D-Api residue (Kobayashi et al. 1996; O’Neill et al. 2004).

Our study of RCKT1 demonstrates that preventing the formation of Sidechain C, a disaccharide seemingly distant from the boron-binding D-Api residue, also disrupts dimer formation. This broadens the prevailing hypothesis that any alteration to the RG-II structure may have a substantial impact on its dimerization and compromise the formation of a normal pectic network in muro. Our CRISPR-mediated strategy provides a basis to test this hypothesis. Generating loss-of-function mutants of RG-II GTs would allow us to create a diverse array of RG-II glycoforms in situ, allowing for an exploration of the relationship between RG-II structure and functionality. Beyond RG-II dimerization, we can also delve into whether structural modifications of RG-II influence potential interaction with other pectic domains and various cell wall components. This would open avenues for us to probe how the pectic network contributes to the mechanical strength and physical properties of the plant primary wall, which play a substantial role in shaping the growth and development of plant cells, tissues, and organs (Braybrook and Peaucelle 2013; Levesque-Tremblay et al. 2015; Yang and Anderson 2020). The formation of a pectic network involves multiple levels of cross-linking, including but not limited to the backbone glycosidic linkages of the 3 pectic domains, the borate ester cross-linking of RG-II, and the calcium cross-linking of HG (Caffall and Mohnen 2009). Although the specific role of RG-II-boron cross-linking in maintaining the physical property of plant cell wall is far from clear, it has been suggested to regulate wall pore size (Fleischer et al. 1999), mechanical strength (Zhou et al. 2017), and extensibility (Cosgrove 2022).

### Challenges to confirm the enzymatic activity of RCKT1 in vitro

Our data have shed light on the role of RCKT1 in the addition of Kdo to RG-II. An in vitro assay for enzymatic activity will be required to increase our understanding of RCKT1's function as an RG-II-specific Kdo transferase. However, this is challenging for several reasons including the difficulties of heterologously expressing a functional RCKT1 enzyme, the availability of the donor substrate CMP-β-Kdo, and the availability of appropriate RG-II glycoforms as acceptor substrates.

It has been challenging to express plant cell wall GTs as stable and active recombinant proteins using bacteria and yeast hosts (Welner et al. 2017; Amos and Mohnen 2019). Our attempts to express RCKT1 using the *Escherichia coli* Origami B strain were not successful. The Human Embryonic Kidney (HEK293) cell system (Moremen et al. 2018) provides an alternative expression platform as it has been used to produce several plant GTs (Urbanowicz et al. 2014; Urbanowicz et al. 2017; Amos et al. 2018; Voiniciuc et al. 2018; Soto et al. 2021; Amos et al. 2022; Engle et al. 2022). However, a bigger challenge arises from the instability of CMP-β-Kdo, the donor substrate for the RCKT1 enzyme. CMP-β-Kdo is rapidly hydrolyzed in aqueous buffers (half-life of ∼34 min), resulting in the formation of CMP and free Kdo (Sugai et al. 1995; Wen et al. 2016). To address this, several groups have generated CMP-β-Kdo in situ using CTP, Kdo, and a CMP-β-Kdo synthetase (KdsB) purified from *E. coli*. This strategy has proven successful in studies focused on bacterial β-Kdo transferases involved in glycolipid biosynthesis (Gronow et al. 2000; Ovchinnikova et al. 2016; Lanz et al. 2021). Finally, an appropriate RG-II glycoform, capable of accepting Kdo from the nucleotide sugar donor CMP-β-Kdo, is required. It is possible that an RG-II lacking Sidechain C could serve as an ideal acceptor for Kdo. The *RCKT1* callus mutants generated in this study could provide this glycoform. Alternatively, Sidechain C could be removed using the bacterial glycanase described by Ndeh et al. (2017). However, the possibility cannot be discounted that a glycoform lacking other sidechains in addition to Sidechain C is the acceptor for Kdo. Tools are now available to modify RG-II structure in vitro and generate different glycoforms in amounts suitable for GT assays (Ndeh et al. 2017). Nevertheless, identifying the appropriate acceptor substrate remains a considerable challenge.

It will also be important to obtain structural information to understand the molecular mechanism of its catalytic activity. This will allow comparison of the ST motifs from the closely related animal STs with the plant RCKT1 to identify potential residues responsible for substrate specificity. Future work will include generating a series of RCKT1 variants for kinetic analysis. It will also be interesting to compare this protein to the previously identified Kdo transferase candidate, AtKDTA, a mitochondrion-localized protein that is thought to have a role in glycosylating an as-yet unidentified lipid signaling molecule (Séveno et al. 2010).

### Advantages of, and potential extensions to, a callus-based gene-editing method

We have demonstrated the utility of our callus-based gene-editing method for generating knockout mutations in 2 GTs that are required for normal plant growth and development. We believe that this approach could serve as a tool for both reverse and forward genetic investigations. One application would be the creation of callus carrying mutations in multiple different GTs. Gene redundancy significantly hampers the characterization of plant GTs (Amos and Mohnen 2019). For example, the GAUT family of *Arabidopsis* has 15 members. Only a few of these have yielded mutants with observable phenotypes that assist in revealing gene function (Sterling et al. 2006; Caffall et al. 2009; Biswal et al. 2018). The multiplexed CRISPR/Cas9 toolbox employed in our study allows the assembly of up to 8 sgRNAs into a single T-DNA vector, a crucial feature that enables the simultaneous editing of multiple gene targets (Lowder et al. 2015).

Our method can also be adapted for use in forward screens to identify candidate GTs involved in specific biological processes, such as cell–cell adhesion. Known cell adhesion mutants exhibit a decreased shoot regeneration phenotype (Johnson et al. 2011). It should be possible to create a library of CRISPR callus mutants for the 75 candidate genes in our target clade and assess their organ regeneration capability. The CRISPR/Cas9 system has been used with rice to generate a large-scale mutant collection (Meng et al. 2017).

Improvements to gene-editing efficiency would enable this method to be more widely implemented. Only one of the three bioinformatically predicted top-performing sgRNAs was able to create CRISPR mutations at the target sites of *GMT1* and *RCKT1*, suggesting the need for experimental prescreening of sgRNAs. Liang et al. (2019) have developed a prescreening method to assess sgRNA efficiency via the *Nicotiana benthamiana* transient expression system that could be applied to select highly efficient sgRNAs for the target genes. This is a rapidly developing area of research, with recent improvements including the use of stronger promoters to drive higher expression of the sgRNAs and Cas9, as well as the utilization of intronized Cas9 (Grützner et al. 2021) or alternative Cas enzymes to enhance editing efficiency in various plant species.

## Materials and methods

### Plant material and incubation conditions

*Arabidopsis* (*A. thaliana*) ecotype Col-0 and *gmt1-3* seeds were surface sterilized for 10 min using a 10% (*v*/*v*) commercial bleach (Clorox, 6.5%, *v*/*v*) in aq. ethanol (100%, *v*/*v*) and then washed twice with 100% ethanol and allowed to air dry overnight. Sterilized seeds were placed on B5 agar media (Supplementary Table S4) and stratified for 3 d at 4°C in the absence of light. The plates were then transferred to a growth chamber for incubation (8,000 lux, 16:8 Light:Dark (L:D), 22°C, 75% humidity). After 10 d, the primary roots were isolated from the seedlings for root transformation as described below. *gmt1-3* allele is as described in Fang et al. (2016). The callus culture of *gmt1-3* was derived from roots as described in Prime et al. (2000).

Calli were maintained by subculturing every 14 d onto freshly prepared callus induction media (CIM) (Supplementary Table S4). The callus selected for subculture should be creamy and white, indicating healthy status. Callus plates were kept in the dark in a growth chamber (25°C, 50% humidity). To grow cell suspension culture in liquid media, callus tissue (∼100 mg) was inoculated into a 1-L flask containing 250-mL liquid CIM and shaken at 100 rpm in the dark at 23°C, a method modified from Prime et al. (2000). Callus from liquid cultures was harvested after 14 d of growth. The tissue was collected into falcon tubes and lyophilized at −50°C for 5 d. Fully lyophilized callus tissue was stored at −80°C for downstream biochemical analysis.

### Screening GTs important for plant cell–cell adhesion by a bioinformatic pipeline

To identify putative GTs involved in the plant cell–cell adhesion process, we set up a bioinformatic pipeline based on the following filtering process.

- Filter I: *Arabidopsis* GT genes were obtained from the CAZy database (http://www.cazy.org/). Genes corresponding to family GT1 proteins were removed since they are largely involved in the glycosylation of small molecules and are unlikely to be involved in cell wall biosynthesis. A total of 441 genes from 49 GT families were identified.
- Filter II: At*GMT1*, which encodes a GIPC mannosyltransferase, was selected as the query gene for coexpression analysis. Coexpression analysis used 113 developmental RNA-seq data sets across 11 tissue and organ types of *Arabidopsis* extracted from ThaleMine (https://bar.utoronto.ca/thalemine/begin.do) (Krishnakumar et al. 2017). Genes with similar expression patterns across the data sets were grouped based on hierarchical clustering using Cluster 3.0 (http://bonsai.hgc.jp/∼mdehoon/software/cluster/). The gene clusters were visualized using TreeView (https://jtreeview.sourceforge.net/), and the clade containing the query gene *GMT1* was extracted.
- Filter III: Genes encoding GTs were localized in Golgi. The subcellular localization of the extracted GTs was obtained by SUBA (https://suba.live/). This step resulted in 75 Golgi-localized candidates as putative GTs most likely to be relevant to plant cell–cell adhesion.

### gRNA design and transformation vector construction

For the 3 sgRNAs designed to target each gene, DNA oligonucleotides were synthesized by Integrated DNA Technologies (Supplementary Table S1) and then annealed and phosphorylated. The phosphorylated sgDNAs were individually cloned into the BsmBI site of the Golden Gate entry vectors pYPQ131A, pYPQ132B, and pYPQ133A obtained from Addgene (https://www.addgene.org/). The 3 gRNA expression cassettes were then cloned into the recipient vector pYPQ143Amp by Golden Gate assembly to create the Gateway entry vector that contains the tri-sgRNA module targeting *AtRCKT1* or *AtGMT1*. The tri-sgRNA module and PcoCas9 were assembled into the destination vector pTKan-p35S::DsRed-p35S::attR1-GW-attR2 by Gateway cloning. This assembly yielded the transformation vectors, pYZ8 and pYZ88, targeting *AtRCKT1* and *AtGMT1*, respectively. Following whole plasmid sequencing verification, the final constructs were transformed into *Agrobacterium tumefaciens* GV3101 for *Arabidopsis* root transformation.

This modular cloning toolkit for CRISPR/Cas9 vector construction was originally developed by Lowder et al. (2015) and modified in this study. The antibiotic selection markers of the Golden Gate recipient vector pYPQ143 and the PcoCas9 entry vector pYPQ150 from the original toolkit were replaced by the ampicillin resistance marker (*AmpR*, GenBank ID: KX682236.1). The resulting vectors, pYPQ143Amp and pYPQ150Amp, can be compatible with the destination vector pTKan-p35S::DsRed-p35S::attR1-GW-attR2 used in this study.

All plasmids and sequence information created in this study are publicly available through the JBEI ICE registry (https://public-registry.jbei.org/login). Construct ID, JBEI registry ID, and construct content of each plasmid are listed in Supplementary Data Set S3.

### *Arabidopsis* root transformation

*A. thaliana* ecotype Col-0 roots were transformed with *A. tumefaciens* GV3101 carrying the CRISPR/Cas9 vectors. The method was modified from Vergunst et al. (1998). Sterilized Col-0 seeds were sown on B5 agar media (Supplementary Table S4) and grown in a growth chamber (8,000 lux, 16:8 L:D, 22°C, 75% humidity). Primary roots were isolated from 10-d-old seedlings using a sterile razor blade and transferred onto CIM (Supplementary Table S4) to incubate for 3 d. *A. tumefaciens* GV3101 carrying the transformation vector was grown for 2 d at 30°C on LB solid media with antibiotics (rifampin, gentamicin, and spectinomycin). A single *A. tumefaciens* colony was inoculated into liquid LB media with antibiotics (100-mg/L rifampin, 10-mg/L gentamicin, and 50-mg/L spectinomycin) and cultured overnight at 30°C with shaking at 200 rpm. *A. tumefaciens* cells were pelleted and then suspended in liquid B5 media (final OD_600_ = 0.1). The isolated primary roots were soaked for 5 min in the *A. tumefaciens* suspension and then sliced into 0.5- to 1-cm explants using a sterile razor blade. The root explants were blot dried on sterile filter paper and plated onto CIM containing D-glucose (1.8 g/L) and acetosyringone (0.1 mM). Root explants were cocultivated with *A. tumefaciens* for 3 d at 25°C under dim light (2,000 lux, 16:8 L:D). The root explants were then washed 4 times with timentin-supplemented (100 mg/L) B5 liquid media and plated onto CIM agar media supplemented with antibiotics (50-mg/L kanamycin and 100-mg/L timentin). After 14 d, the explants were transferred to fresh CIM containing kanamycin and timentin. Calli resistant to kanamycin were collected and subcultured every 14 d.

### CRISPR editing analysis of the transgenic callus mutants

Callus tissue was sampled from each independent transformation event, flash frozen in liquid nitrogen, and then milled with metallic beads using a TissueLyser (Qiagen) for subsequent genomic DNA (gDNA) extraction. Genomic DNA was extracted from callus using the QIAGEN DNeasy Plant Mini Kit. Primers that amplified the genomic region of the gRNA target sites were used to genotype the PCR amplicon (Supplementary Table S5). PCR amplicons were column purified using the Zymo Research DNA Clean & Concentrator Kit, and Sanger sequenced by Azenta (https://www.azenta.com/). Decodr v3.0 (https://decodr.org/) was used to deconvolute the sequenced amplicon for mutation analysis.

### GIPC purification and analysis by TLC

GIPC analysis by TLC was performed as described by Jing et al. (2021). Lyophilized callus tissue (∼0.5 g dry weight) harvested from 3 distinct liquid cultures was combined and ground to a powder with metallic beads using a TissueLyser (Qiagen). Sphingolipids were extracted from the ground tissue first using the lower layer of the isopropanol/hexane/water (55:20:25, *v*/*v*/*v*), followed by deesterification with 4 mL of 33% (*w*/*v*) methylamine solution in ethanol/water (7:3, *v*/*v*) as previously described (Markham and Jaworski 2007). The water used in this experiment is HPLC grade. To enrich the GIPCs, the dried extracts were suspended in 1 mL of enrichment solution (chloroform/ethanol/ammonia/water [10:60:6:24, *v*/*v*/*v*/*v*]) and kept overnight at room temperature with agitation (2 rpm). The GIPCs were then isolated using a weak anion exchange SPE cartridge (Strata X-AW; Phenomenex) as previously described (Mortimer et al. 2013). GIPCs bound to the cartridge were eluted with 3 mL of enrichment solution and dried under a flow of nitrogen gas or a stream of clean dry air. The dried GIPCs were suspended in Solution A (chloroform/methanol/[4 M ammonium hydroxide in 1.8 M ammonium acetate] [9:7:2, *v*/*v*/*v*]) and separated by TLC. The separations were performed on high-performance TLC Silica gels on glass plates (Merck) with Solvent A as the mobile phase. GIPCs were stained with primuline (10 mg in 100 mL acetone/water [8:2, *v*/*v*]) and visualized under a fluorescent blue light (460 nm) (Skipski 1975).

### GIPC glycan profiling by HPAEC-PAD

Purified GIPCs were treated for 1 h at 120°C with 2 M TFA (1 mL), cooled on ice, and dried overnight using a vacuum concentrator. The residues were dissolved (2- to 5-fold) in HPLC grade water and the liberated monosaccharides were analyzed by HPAEC-PAD as previously described (Fang et al. 2016).

### Preparation of plant cell walls as AIR

AIR was prepared as previously described (Mortimer et al. 2013). Briefly, lyophilized callus tissue was milled into a fine powder and then suspended in 96% (*v*/*v*) ethanol. The suspension was kept at 70°C for 30 min and then cooled and centrifuged (15 min, 18,500 rcf). The pellet was then suspended in ethanol (96%, *v*/*v*), vortexed, and centrifuged again (15 min, 18,500 rcf). The pellet was then suspended in methanol:chloroform (2:3, *v*/*v*) and kept overnight at 4°C with agitation (300 rpm). Following centrifugation (15 min, 18,500 rcf), the pellet was treated for 1 h at 4°C methanol:chloroform (2:3, *v*/*v*) (1 h, 300 rpm). Following centrifugation (15 min, 18,500 rcf), the pellet was washed with the following ethanol series: 100% (*v*/*v*), 65% (*v*/*v*), 70% (*v*/*v*), 80% (*v*/*v*), and 100% (*v*/*v*). Each wash involved a 5-min centrifugation (18,500 rcf) and removal of the supernatant in between, with a thorough vortex to suspend the pellet. The resulting AIR was dried at 50°C overnight in a vacuum concentrator or oven.

Starch was removed by treating the AIR (∼10 mg) for 10 min at 75°C with agitation at 300 rpm with *α*-amylase (3 U/mL in 50 mM MOPS, pH 7.3). Pullulanase (1 U/mL) and amyloglucosidase (3 U/mL in 200 mM sodium acetate, pH 4.5) were then added, and the suspensions were kept for a further 2 h at 40°C (with agitation at 300 rpm). Following centrifugation, the pellet was washed with the following ethanol series: 100% (*v*/*v*), 70% (*v*/*v*), and 70% (*v*/*v*). The destarched AIR was dried overnight at 30°C in a vacuum concentrator.

### Cell wall monosaccharide composition analysis

Noncellulosic sugars were liberated from destarched AIR by TFA hydrolysis for 1 h at 120°C. The supernatant containing the liberated monosaccharides was collected after centrifugation. Residual monosaccharides in the remaining pellet were further extracted using water and combined with the supernatant. The combined supernatant was dried overnight at 30°C in a vacuum concentrator. The residue was suspended in HPLC grade water and filtered through a nitrocellulose filter (0.45 *μ*M). The noncellulosic sugars in the filtrate were diluted (4- to 5-fold) with water and filtered through a nitrocellulose filter (0.45 *μ*M) prior to HPAEC-PAD analysis (Fang et al. 2016).

The TFA-insoluble AIR pellet was then subjected to Saeman hydrolysis to hydrolyze cellulose. The pellet was suspended in 72% (*v*/*v*) sulfuric acid and kept for 1 h at room temperature. Water was added to dilute the sulfuric acid to 1 M, and the suspensions were then heated for 3 h at 100°C. The suspensions were then neutralized by adding barium carbonate and centrifuged. The supernatant was dried overnight at 30°C in a vacuum concentrator. The residue was suspended in water and filtered through a nitrocellulose filter (0.45 *μ*M). The sugars in the filtrate were diluted with water and filtered prior to HPAEC-PAD analysis. The water used in this experiment is HPLC grade.

### Isolation of RG-II from AIR of controls and the *rckt1* mutants

The callus AIR (500 to 700 mg) extracted from 3 distinct liquid cultures was combined and suspended in NaOAc (50 mM, pH 5.0) at a concentration of 25 mg/mL and then treated for 24 hr at 37 °C with 5-U/g EPG and 10-U/g pectin methyl esterase (PME) with shaking at 150 rpm. The EPG solubilized material and the insoluble residue were separated by filtration through a nylon mesh (100 *μ*m). The insoluble material retained on the mesh was treated again with EPG and then filtered as before. The EPG-soluble materials were obtained by combining the filtrates after the 2 treatments. The filtrates were then fractionated by preparative SEC on a column Superdex Increase 75 (10/300 GL, Cytiva, USA, column L × I.D. 30 cm × 10 mm, 9 *μ*m), with refractive index detection. The column was eluted with ammonium formate (50 mM, pH 5.0) at 0.5 mL/min. Fractions enriched in RG-II were collected manually, dialyzed (3500 Dalton MWCO) against deionized water, and freeze dried.

### RG-II glycosyl residue composition analyses

To specifically detect Kdo and Dha, the glycosyl residue of RG-II was analyzed as their TMS methyl glycoside derivatives by GC-EI-MS as described in Barnes et al. (2021). In general, RG-II (100 *μ*g) was suspended in 300-*μ*L methanolic HCl (1 M) in screw-top glass tubes secured with Teflon-lined caps and heated at 80 °C for 18 hr. After cooling to room temperature, the solutions were concentrated to dryness under the nitrogen stream. The released methyl glycosides and methyl glycoside methyl esters were then reacted for 30 min at 80 °C with Tri-Sil (Thermo Fisher, USA). GC-EI-MS analysis of the TMS methyl glycosides was performed on an Agilent 7890A GC interfaced to an Agilent 5975C mass selective detector, with a Supelco Equity-1 fused silica capillary column (30 m × 0.25 mm ID).

### Preparation of RG-II monomer for NMR spectroscopic analysis

The RG-II dimer was converted to the monomer as previously described (Barnes et al. 2021). Briefly, the RG-II enriched material obtained by SEC was treated for 1 h at room temperature with HCl (0.1 M). The solution was then dialyzed (3500 Dalton MWCO) against deionized water and freeze dried.

### ^1^H-NMR and 2D TOCSY spectroscopy of RG-II monomer

RG-II (1 to 2 mg) was dissolved in D_2_O (0.2 mL, 99.9%; Cambridge Isotope Laboratories, Tewksbury, MA, USA) and placed in a 3-mm NMR tube (Barnes et al. 2021). Spectra were obtained using a Bruker NMR spectrometer (Agilent Technologies) operating at 900 MHz using a 5-mm cold probe and a sample temperature of 25°C. The 1D ^1^H and 2D TOCSY spectra were recorded using standard Bruker pulse programs. Chemical shifts of the RG-II glycosyl residue were measured relative to internal D_2_O (δH 4.707). Data were processed using MestReNova software (Mestrelab Research S.L., Santiago de Compostela, Spain).

### Statistical analysis

One-way ANOVA was used for statistical analysis.

### Accession numbers

Sequence data from this article can be found at TAIR under accession numbers At3g55830 (*AtGMT1*), At1g08660 (*AtRCKT1*), AT3G48820 (*AtSIA2*), and AT1G08280 (*AtGALT29A*).

## Supplementary Material

kiae259_Supplementary_Data

## Data Availability

The data supporting the findings of this study are available within the paper and its supplementary data.

## Supplementary data

The following materials are available in the online version of this article.

**Supplementary Figure S1**. Tailored CRISPR/Cas9 platform for GT editing in *Arabidopsis*.

**Supplementary Figure S2**. GIPC analysis using TLC.

**Supplementary Figure S3**. GC-EI-MS total ion current profiles of the TMS methyl ester methyl glycoside derivatives of the monosaccharides generated from RG-II.

**Supplementary Table S1**. sgRNA sequences for the target genes.

**Supplementary Table S2**. Mannose content of the GIPCs isolated from *Arabidopsis* callus tissue.

**Supplementary Table S3**. The abundance of the RG-II dimer and monomer in the material released by EPG and PME treatment of the AIR.

**Supplementary Table S4**. Composition of *A. thaliana* tissue culture media.

**Supplementary Table S5**. Sanger sequencing primers for genotyping CRISPR callus mutants.

**Supplementary Data Set S1**. Putative GTs coexpressed with *AtGMT1* and presumably localized to Golgi.

**Supplementary Data Set S2**. Cell wall polysaccharides raw data of *rckt1* callus mutants.

**Supplementary Data Set S3**. Expression vectors and parts for their assembly.

## Dive Curated Terms

The following phenotypic, genotypic, and functional terms are of significance to the work described in this paper:

GAUT4 Gramene: AT5G47780
GAUT4 Araport: AT5G47780

mur1 Gramene: AT3G51160
mur1 Araport: AT3G51160

CMP CHEBI: CHEBI:17361

water CHEBI: CHEBI:15377

GALT29A Gramene: AT1G08280
GALT29A Araport: AT1G08280

RCKT1 Gramene: https://www.arabidopsis.org/locus?key=29154
RCKT1 Araport: https://www.arabidopsis.org/locus?key=29154
