# Supplementary material for: Putative rhamnogalacturonan-II glycosyltransferase identified through callus gene editing which bypasses embryo lethality
Source: Plant Physiol. 2024 May 13;195(4):2551–65. doi: 10.1093/plphys/kiae259 (PMC11288761; doi:10.1093/plphys/kiae259)
Supplement: kiae259_Supplementary_Data [file kiae259_supplementary_data.zip › kiae259_Supplementary_Data.pdf]

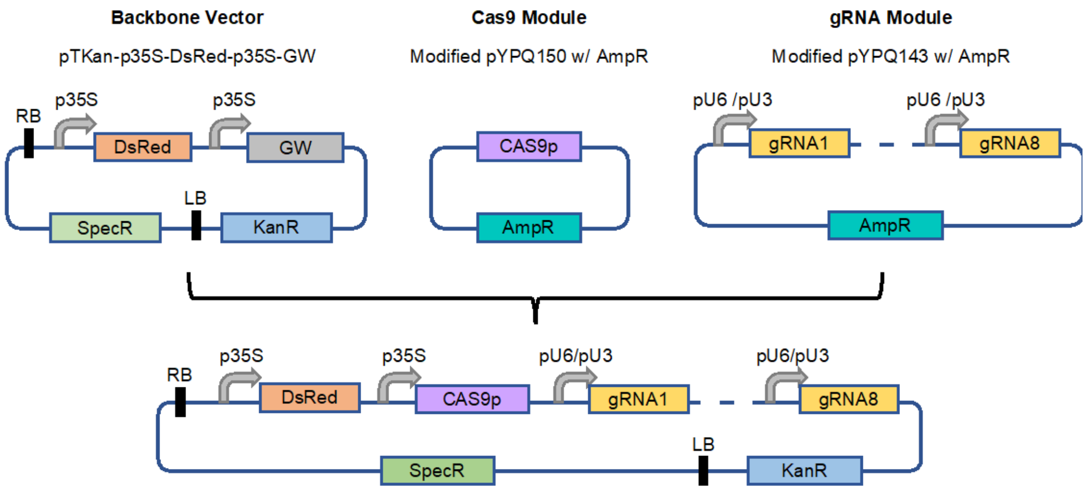

Supplementary Figure S1. Tailored CRISPR/Cas9 platform for GT editing in Arabidopsis. The assembly procedure of CRISPR/Cas9 T-DNA vector is adapted from (Lowder et al. 2015)**.** Three modules are required for this platform: a T-DNA destination vector providing 35S promoter for Cas9 expression; a Cas9 entry vector; a gRNA entry vector that contains up to eight gRNA expression cassettes. The T-DNA transformation vector can be made by the Multisite Gateway recombination to assemble the Cas9 and the gRNA modules into the T-DNA destination vector. The module containing multiple gRNA expression cassettes is made by a two-step Golden Gate cloning as described in (Lowder et al. 2015). SpecR, spectinomycin resistance marker; KanR, kanamycin resistance marker; DsRed, DsRed fluorescence reporter; GW, Gateway recombination sites; 35S, CaMV 35S promoter; LB, left border region; RB, right border region; AmpR, ampicillin resistance marker; pU6, Arabidopsis ubiquitin6 promoter; pU3, Arabidopsis ubiquitin3 promoter.

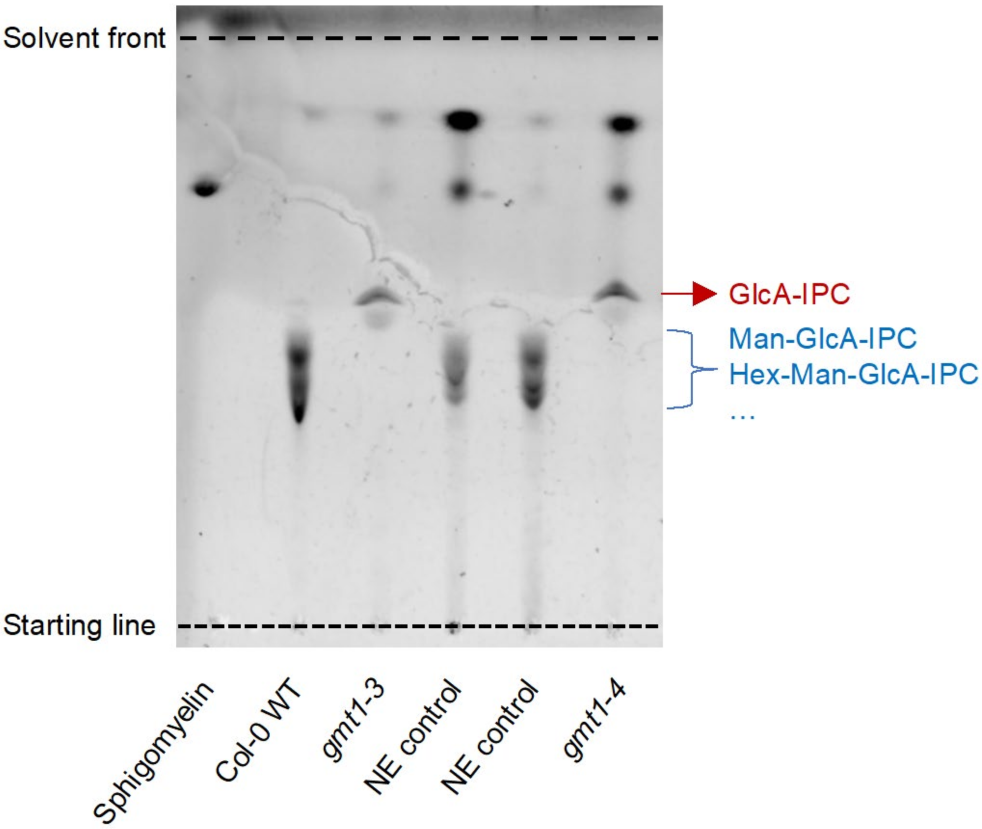

Supplementary Figure S2. GIPC analysis using TLC. GIPCs were extracted from Arabidopsis callus tissue of Col-0 wild-type, *gmt1-3*, NE control, and *gmt1-4*. The enriched GIPCs and sphingomyelin standard were run on a TLC plate and stained with Primuline before imaging. GlcA-IPC is indicated by the red arrow, while the cluster of highly glycosylated GIPC species (e.g. Man-GlcA-IPC and Hex-Man-GlcA-IPC) is indicated by the blue bracket.

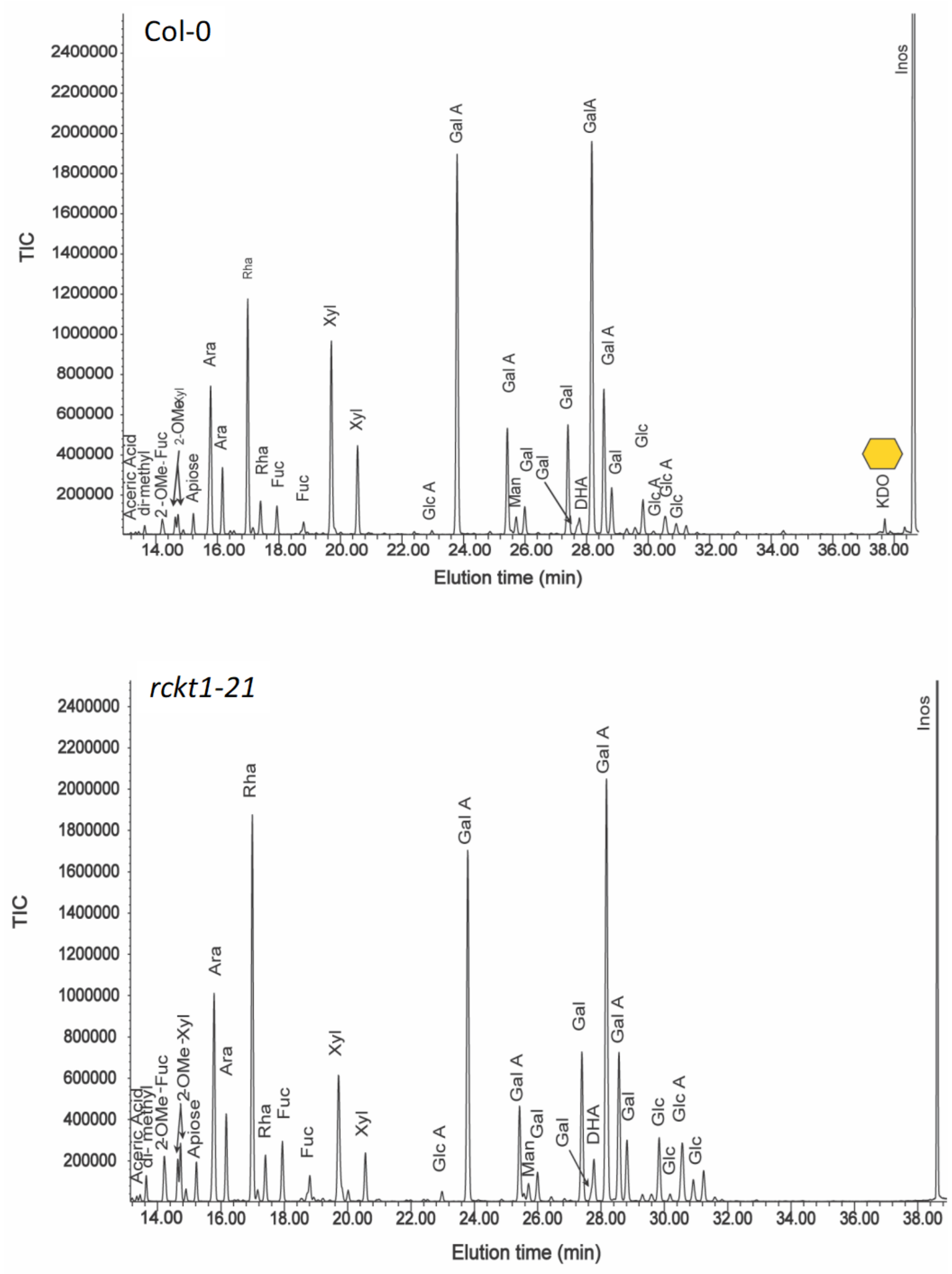

Supplementary Figure S3. GC-EI-MS total ion current profiles of the trimethylsilyl methyl-ester methyl glycoside derivatives of the monosaccharides generated from RG-II. The GC-EI-MS total ion current (TIC) profile of the TMS derivatives generated from RG-II of **A**, Col-0 wild-type, **B**, *rckt1-21*. The identity of the monosaccharide derivative in each peak is shown. The peak eluting at ~ 39 min is the TMS derivative of Myo-inositol used as an internal standard. The peak of 3-deoxy-D-manno-octulosonic acid (Kdo, yellow hexagon) is present in **A**, but absent in **B**.

Supplementary Table S1. Single guide RNA (sgRNA) sequences for the target genes.

Target gene	gRNA sequence (5’ -> 3’)	
GMT1	gRNA1	TACGCAAGTTCGTGACGGCG
	gRNA2	GTTCAATCGCCGTCGCAGAT
	gRNA3	GCATGAGGTTGAGCTGAGAT
RCKT1	gRNA1	GTCCAGCGTTCAACAGTGCG
	gRNA2	CTGAAGGCACTAACAGTACT
	gRNA3	TGTACGCCCTGATGGGTGGT

Supplementary Table S2. **Mannose content of the GIPCs isolated from Arabidopsis callus tissue.** The GIPCs were extracted from a pooled collection of callus tissue obtained from three independently grown liquid culture for each line. Following extraction, the enriched GIPC fraction was hydrolyzed by TFA to liberate monosaccharides within their glycan headgroups. The derived monosaccharides were quantified by HPAEC-PAD. The mannose quantity was normalized to the dry weight (DW) of callus tissue.

**Mannose content**	Col-0	*gmt1-3*	NE control	*gmt1-4*
(mg/g DW)	7	1	6	2

Supplementary Table S3. The abundance of the RG-II dimer and monomer in the material released by endopolygalacturonase (EPG) and pectin methyl esterase (PME) treatment of the alcohol-insoluble residue (AIR).

Callus line	Released by EPG and PME treatment of AIR	
	Dimer	Monomer
	% of total RG-II[a]	
Col-0	70	30
NE Control	80	20
rckt1-9	26	74
rckt1-21	22	78

[a]The abundance of the dimer and monomer was determined by size-exclusion chromatography (SEC) with RI detection.

Supplementary Table S4. Composition of *Arabidopsis thaliana* tissue culture media.

Components		B5	Callus induction media (CIM)[a]
Gamborg B-5 Basal Medium		3.2 g/L	3.2 g/L
D-Glucose			20 g/L
MES		0.5 g/L	0.5 g/L
Difco Agar, Bacteriological		9 g/L	9 g/L
Phytohormones	2,4-D		0.5 mg/L
	Kinetin		0.05 mg/L

[a]For co-cultivating Col-0 root explants with *A. tumefaciens*, CIM media with reduced glucose content (1.8 g/L) is supplemented with 0.1mM acetosynringone. For selecting transgenic callus line, CIM media is supplemented with 50 mg/L Kanamycin and 100 mg/L Timentin.

Supplementary Table S5. Sanger sequencing primers for genotyping CRISPR callus mutants.

**Primers**	**Sequence (5' -> 3')**
GMT1 Seq Primer_F	CACAGTTGACTTCTGAGACG
GMT1 Seq Primer_R	CAGAACGTGTGTGTGTGTAC
RCKT1 Seq Primer_F	GAGATCTCACCAAGCTGGCC
RCKT1 Seq Primer_R	GTCGAAGTGTCGGTAAACTC
