# Supplementary material for: Putative rhamnogalacturonan-II glycosyltransferase identified through callus gene editing which bypasses embryo lethality
Source: Plant Physiol. 2024 May 13;195(4):2551–65. doi: 10.1093/plphys/kiae259 (PMC11288761; doi:10.1093/plphys/kiae259)
Supplement: kiae259_Supplementary_Data [file pp.00260.2024-s06.pdf › pp.00260.2024-s06.pdf]

Plant Physiology®

Identification of a putative rhamnogalacturonan-II glycosyltransferase via callus gene editing bypasses embryo lethality

Yuan Zhang, Deepak Sharma, Yan Liang, Nick Downs, Fleur Dolman, Kristen Thorne, Ian Black, Jose Pereira, Paul Adams, Henrik Scheller, Malcolm O'Neill, Breeanna Urbanowicz, and Jenny Mortimer

Review Timeline:

Submission Date:	21-Feb-2024
Editorial Decision:	25-Mar-2024
Revision Received:	09-Apr-2024
Accepted:	10-Apr-2024

25-Mar-2024

Dr. Jenny C Mortimer
University of Adelaide
Glen Osmond, SA
Australia

RE: A putative rhamnogalacturonan-II CMP-β-Kdo transferase identified using gene editing in calli to bypass embryo lethality

Dear Dr. Jenny Mortimer:

We are pleased to accept your manuscript for publication in the Plant Physiology. This acceptance is contingent on revision based on the comments of our reviewers. In particular, please consider addressing ALL the minor suggestions of both reviewers!

Please highlight all changes and include a detailed annotation to changes to the text, with line numbers, and noting your responses to the comments.

To submit your revised manuscript, click:

Link Not Available

If you cannot return the revised manuscript within 8 weeks of receipt, please let us know. Otherwise, we will assume that you have elected not to revise the manuscript and are withdrawing it.

Thank you for allowing us to review your work. We look forward to hearing from you soon.

Sincerely,

Board of Editors Plant Physiology

-- Reviewer comments:
Reviewer #1 (Comments for the Author):

The Zhang et al. manuscript uses a novel strategy to identify a gene encoding a protein that putatively adds 3-deoxy-D-manno-octulosonic acid (Kdo) to Rhamnogalacturonan II (RG-II). This new strategy may well have relevance for efforts to identify other genes that encode essential proteins, especially proteins involved in cell wall biosynthesis. Briefly, the authors argue that because RG-II is essential for higher plant function, it has not been possible to isolate mutants with defects in RG-II. They circumvent this obstacle by using CRISPR/Cas-9-mediated gene editing to isolate Arabidopsis callus cells containing mutations in putative genes involved in RG-II biosynthesis. The callus cells can grow, presumably because the functions of RG-II are not needed in these cells. Using a candidate gene approach, the create mutations in MGB2, a gene in the family of GT29 glycosyltransferases. They isolate RG-II from the mutant callus lines and provide several lines of evidence that it specifically lacks Kdo.
The experiments reported in the manuscript were clearly and logically explained. For the most part, the conclusions presented in the manuscript were supported by the results. I have no major concerns about the manuscript, or the data presented in it. However, I do have several minor suggestions and comments that the authors should consider.
The first suggestion is to create a different title for the manuscript that will be more understandable by readers of Plant Physiology who are not cell wall experts. Specifically, I am concerned that the use of the enzyme name, i.e., "...CMP-beta-Kdo transferase..." will not be understood by many readers. It should be possible to create a title that avoids the use of the name, which includes unexplained abbreviations.
In addition, I have a few other comments about minor issues that should be addressed.

1. In some places the authors make specific claims about earlier work with no citations; they should provide citations to document these points. These are present in lines 51 -53 where they say: "Mutations that result in alterations to RG-II structure also affect crosslinking and may be lethal or severely impair growth." Another example is lines 60-61 where they say: "Plants carrying mutations in genes that affect RG-II synthesis and structure typically exhibit severe growth defects." In line 134, they say: "No homozygous mutants of GALT29A, RCKT1, (MGP2), and SIA2 have been isolated,...". How do they know this?
2. In line 149, the abbreviation NHEJ is introduced, but I could not find where it was used again later. Should it be removed?
3. In line 249, is the word "conclusively" needed?
4. Supplemental Figure 2 was difficult to interpret. It would be helpful to readers if the various compounds separated by TLC could be identified. If spots are not specific compounds, such as sphingomyelin, then it would be useful to readers to explain

what collection of compounds are present in the various spots.

Reviewer #2 (Comments for the Author):

In this article entitled « A putative rhamnogalacturonan-II CMP-β-Kdo transferase identified using gene editing in calli to bypass embryo lethality », the authors described a CRISPR-mediated transformation to generate callus with loss-of-function of glycosyltransferases. They first validated this approach on the mannosyl-transferase 1 involved in the biosynthesis of glycosylinositol phosphorylceramide and then on MGP2/RCKT1, a putative GT29 sialyltransferase-like candidate protein. Authors showed that rckt1 mutants lack Kdo in their sugar compositions. Proton 1 and 2D nmr of monomeric RG-II clearly demonstrated that these mutants are devoid of the Rha-(1→5)-kdo side chain but not of the homologous Ara-(1→5)-Dha side chain. Together with in silico data, these results demonstrate that RCKT1 is a CMP-Kdo transferase although additional biochemical assays cannot be performed for confirmation (instability of CMP-Kdo). Moreover, RG-II dimerisation is strongly affected which is consistent with the lethality of homozygous RCKT1 mutant plants as previously reported for plant mutants deficient in RG II-specific GT, RG-II-specific nucleotide-sugars or their Golgi transporters. This also supports the hypothesis that any alteration to the RG-II structure strongly impacts its in muro dimerization although Kdo is not directly involved in the borate cross linking.

The CRISPR-mediated strategy describes in this article provides a new and promising way to generate loss-of-function mutants of RG-II bypassing embryo lethality and thus, a new approach to study RG II structure-function relationship.

I recommend this article for publication after considering the following minor comment on cited references :

Line 284 : the first paper describing GDP-L-Gal deficiency is : Voxeur et al., (2011) Silencing of the GDP-D-mannose 3,5-epimerase affects the structure and cross-linking of the pectic polysaccharide rhamnogalacturonan II and plant growth in tomato. Journal of Biological Chemisty 286, 8014-8020.

Response to the Reviewers comments Re: PP2024-RA-00260

We thank the reviewers for the time taken to carefully review our manuscript, and their supportive comments. Each made minor suggestions, which we have now fully addressed, as detailed below:

<u>Response to the comments of Reviewer #1:</u>

1. **Comment by Reviewer 1:** The first suggestion is to create a different title for the manuscript that will be more understandable by readers of Plant Physiology who are not cell wall experts. Specifically, I am concerned that the use of the enzyme name, i.e., "...CMP-beta-Kdo transferase..." will not be understood by many readers. It should be possible to create a title that avoids the use of the name, which includes unexplained abbreviations.

Our response: We agree with the Reviewer, and we have changed the title to the following:

"Identification of a putative rhamnogalacturonan-II glycosyltransferase through gene editing of callus bypasses embryo lethality"

2. **Comment by Reviewer 1:** In some places the authors make specific claims about earlier work with no citations; they should provide citations to document these points.

1) These are present in lines 51 -53 where they say: "Mutations that result in alterations to RG-II structure also affect crosslinking and may be lethal or severely impair growth."

Our response: Apologies for these oversights. The following highly relevant references have been added to support this statement.

(O'Neill et al., 2001; Delmas et al., 2008; Reboul et al., 2011; Pabst et al., 2013; Rautengarten et al., 2016; Qi et al., 2017; Sechet et al., 2018)

2) Another example is lines 60-61 where they say: "Plants carrying mutations in genes that affect RG-II synthesis and structure typically exhibit severe growth defects."

Our response: We believe that sufficient evidence evidence and the corresponding references are now provided in the same paragraph to support this claim.

3) In line 134, they say: "No homozygous mutants of GALT29A, RCKT1, (MGP2), and SIA2 have been isolated,...". How do they know this?

Our response: The following references have been added to support this statement. (Deng et al., 2010; Dumont et al., 2014)

2. In line 149, the abbreviation NHEJ is introduced, but I could not find where it was used again later. Should it be removed?

Our response: We have deleted this abbreviation, “(NHEJ)”.

3. In line 249, is the word "conclusively" needed?

Our response: We have deleted this word, "conclusively".

4. Supplemental Figure 2 was difficult to interpret. It would be helpful to readers if the various compounds separated by TLC could be identified. If spots are not specific compounds, such as sphingomyelin, then it would be useful to readers to explain what collection of compounds are present in the various spots.

Our response: Thank you for this suggestion. We have now labeled the GIPC spots clearly on Supplemental Figure 2, and revised the figure legend accordingly.

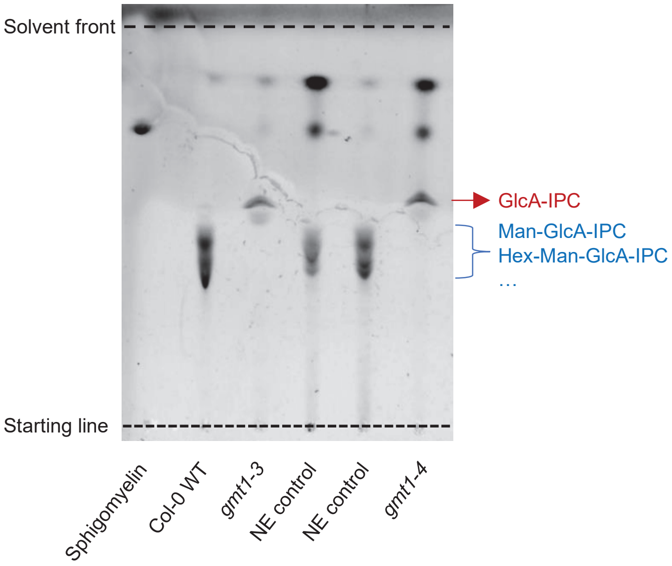

Supplementary Figure 2. GIPC analysis using TLC. GIPCs were extracted from Arabidopsis callus tissue of Col-0 wild-type, *gmt1-3*, NE control, and *gmt1-4*. The enriched GIPCs and sphingomyelin standard were run on a TLC plate and stained with Primuline before imaging. GlcA-IPC is indicated by the red arrow, while the cluster of highly glycosylated GIPC species (e.g. Man-GlcA-IPC and Hex-Man-GlcA-IPC) is indicated by the blue bracket.

Response to the comments of Reviewer #2:

1. Line 284 : the first paper describing GDP-L-Gal deficiency is : Voxeur et al., (2011) Silencing of the GDP-D-mannose 3,5-epimerase affects the structure and cross-linking of the pectic polysaccharide rhamnogalacturonan II and plant growth in tomato. Journal of Biological Chemisty 286, 8014-8020.

Our response: We thank the reviewer for catching this error, and we apologise for the oversight. The suggested references have been added.

10-Apr-2024

Dr. Jenny C Mortimer
University of Adelaide
Glen Osmond, SA
Australia

MSID: PP2024-RA-00260R1
MS TITLE: Identification of a putative rhamnogalacturonan-II glycosyltransferase via callus gene editing bypasses embryo lethality

Dear Jenny:

I am pleased to inform you that your revised manuscript "Identification of a putative rhamnogalacturonan-II glycosyltransferase via callus gene editing bypasses embryo lethality" has been accepted for publication in Plant Physiology. Your manuscript will be checked for consistency with journal formatting and image standards by one of our Science Editors, who will contact you shortly. They may have a number of queries and suggestions for you. Please keep in mind that they work to increase the online visibility of your article and its attraction for readers from the widest of backgrounds. You will have the opportunity to review any revisions, and I hope you will work constructively with them to get the most out of your research. You will also have the opportunity to review all changes when you receive the author proofs for your article.

Once validated for publication, your article will be sent to a Science Editor for a final review of your manuscript and source files. After your final files are uploaded, your article will appear in the next available issue and the online Preview version of your article will be posted after your signed Copyright and Checklist forms and final source files are received.

Please be sure to include the following in your manuscript file:
Harvard style citations
List of all author contributions and funding information at the beginning of manuscript after the titles and author list.
List all supplemental materials associated with the article, with titles, at the end of the ms, before the references and acknowledgments section.
Complete Figure Legends for all figures appearing in the article listed before the Literature Cited.

Thank you and congratulations on your Plant Physiology paper!

Sincerely,
Board of Editors Plant Physiology

=====
FIRST AUTHOR PROFILES
=====

We recognize the hard work that goes into being First Author of a Plant Physiology paper by publishing a "First Author Feature" for each First Author alongside your paper.

If the first author(s) would like to submit information for a feature on Facebook and Twitter, fill out the form by clicking on the link below:

Plant Physiology First Author Feature Form: https://forms.office.com/r/ZBCGZfhm8F
If you cannot access the form through the link above, please contact Rachel at rbelsky@aspb.org.

To upload an image of yourself to accompany your profile, please put your photo in the correct journal folder at the link below. You must label your image: Author First Name Last Name Article Title.

First Author Profile Image Dropbox: https://www.dropbox.com/scl/fo/xz029p7qjr2jml2rp2kmq/h?dl=0&rlkey=6fkxcaqmxx35ap152b2osy1fz

=====
IMPORTANT REMINDER: PEER REVIEW REPORTS
=====

If you opted to publish a peer review report along with your article during the original submission process, it will be prepared by the editorial staff and publicly posted with your manuscript, inside the zip file that contains any other supplemental material. As a reminder, the peer review report is a public record of all comments from editors and reviewers, as well as your prior responses, as you received them in the decision letters for each draft of your manuscript. If you agreed to publish this report and have changed your mind, or are not sure if you selected this option, please contact the editorial office as soon as possible before signing the license agreement from our publisher.

-- Reviewer comments:
